# Von Hippel–Lindau disease: insights into oxygen sensing, protein degradation, and cancer

**DOI:** 10.1172/JCI162480

**Published:** 2022-09-15

**Authors:** William G. Kaelin

**Affiliations:** Howard Hughes Medical Institute, Chevy Chase, Maryland, USA and Department of Medical Oncology, Dana-Farber Cancer Institute and Brigham and Women’s Hospital, Harvard Medical School, Boston, Massachusetts, USA.

## Abstract

Germline loss-of-function mutations of the VHL tumor suppressor gene cause von Hippel–Lindau disease, which is associated with an increased risk of hemangioblastomas, clear cell renal cell carcinomas (ccRCCs), and paragangliomas. This Review describes mechanisms involving the VHL gene product in oxygen sensing, protein degradation, and tumor development and current therapeutic strategies targeting these mechanisms. The VHL gene product is the substrate recognition subunit of a ubiquitin ligase that targets the α subunit of the heterodimeric hypoxia-inducible factor (HIF) transcription factor for proteasomal degradation when oxygen is present. This oxygen dependence stems from the requirement that HIFα be prolyl-hydroxylated on one (or both) of two conserved prolyl residues by members of the EglN (also called PHD) prolyl hydroxylase family. Deregulation of HIF, and particularly HIF2, drives the growth of VHL-defective ccRCCs. Drugs that inhibit the HIF-responsive gene product VEGF are now mainstays of ccRCC treatment. An allosteric HIF2 inhibitor was recently approved for the treatment of ccRCCs arising in the setting of VHL disease and has advanced to phase III testing for sporadic ccRCCs based on promising phase I/II data. Orally available EglN inhibitors are being tested for the treatment of anemia and ischemia. Five of these agents have been approved for the treatment of anemia in the setting of chronic kidney disease in various countries around the world.

Von Hippel–Lindau (VHL) disease affects approximately 1 in 35,000 people worldwide and presents clinically as an autosomal dominant hereditary cancer syndrome (1). The classical tumors seen in this disorder are blood vessel tumors called hemangioblastomas of the retina, cerebellum, and spinal cord; clear cell renal cell carcinomas (ccRCCs); and sympathetic nervous system tumors called paragangliomas. Paragangliomas arising in the adrenal gland are referred to a pheochromocytomas. Patients with VHL disease usually harbor a germline loss-of-function *VHL* mutation or, less frequently, have a mosaic loss-of-function *VHL* mutation that was acquired after conception. Tumors develop when the remaining wild-type *VHL* allele is mutated or lost.

ccRCC is the most common form of kidney cancer, which is one of the ten most common cancers in the developed world (2). Consistent with the knowledge that germline *VHL* mutations predispose to ccRCC, biallelic *VHL* mutations or, less frequently, hypermethylation are very common in sporadic ccRCC (3, 4). Somatic *VHL* mutations have also been described in sporadic hemangioblastomas and paragangliomas (5, 6). In short, the *VHL* gene behaves as a classical Knudson two-hit tumor suppressor gene.

VHL disease is not genetically heterogeneous insofar as all patients who phenotypically have VHL disease are known or can be presumed to harbor a *VHL* mutation. On the other hand, there are striking genotype-phenotype correlations in VHL disease (3). VHL families are said to have type 1 disease if they display a low risk of paraganglioma and type 2 disease if they display a high risk of paraganglioma. Type 2 disease can be subdivided into type 2A disease (low risk of ccRCC), type 2B disease (high risk of ccRCC), and type 2C disease (paraganglioma only). Interestingly, almost all *VHL* mutations associated with type 2 disease are missense mutations, while *VHL* mutations associated with type 1 disease include true null *VHL* mutations and grossly destabilizing *VHL* missense mutations.

Some *VHL* mutations have been linked to familial polycythemia rather than to VHL disease. These individuals carry homozygous (or, less commonly, compound heterozygous) hypomorphic *VHL* alleles (Chuvash polycythemia). In contrast, VHL disease patients carry a wild-type allele and a defective *VHL* allele. Accordingly, all the somatic cells (including cells capable of producing erythropoietin) in Chuvash polycythemia patients are hypomorphic with respect to pVHL function. VHL disease patients are initially normal, because there is no evidence of *VHL* haploinsufficiency.

The *VHL* gene encodes two different proteins by virtue of alternative, in-frame, start codons (7–9). The longer protein contains 213 amino acid residues, with the shorter version beginning at methionine located at codon 54. For simplicity, I will refer to both isoforms as pVHL, partly because the long and short forms share key biochemical functions and are both capable of suppressing tumor growth.

pVHL is primarily a cytosolic protein, but can dynamically shuttle in and out of the nucleus (10–12). pVHL serves as the substrate recognition component of a multisubunit ubiquitin ligase that includes elongin B, elongin C, cullin 2, and Rbx1 (3). A number of substrates for this ubiquitin ligase have been reported, although the substrate most clearly linked to pVHL’s tumor suppressor activity is the hypoxia-inducible factor (HIF) transcription factor. In addition, pVHL has been reported to control the activity of specific kinases, such as AKT (13) and casein kinase 2 (14), in a ubiquitin-independent manner.

HIF consists of a labile α subunit (for example, HIF1α) that is normally degraded when oxygen is plentiful and a constitutively stable β subunit (for example, HIF1β, which is more commonly called ARNT1) (15). The pVHL ubiquitin ligase is responsible for the oxygen-dependent ubiquitylation, and hence degradation, of HIFα (15). In the presence of oxygen HIFα is prolyl-hydroxylated on one (or both) of two conserved prolyl residues by members of the EglN (also called PHD) prolyl hydroxylase family, which thereby generates a high-affinity pVHL binding site (16–21) (Figure 1). EglN1 (also called PHD2) is the primary regulator of HIFα stability under normal conditions in the cells and tissues examined to date, with EglN2 and EglN3 serving to further fine-tune the hypoxic response (22, 23). The EglNs have low oxygen affinities, and as a result they are sensitive to decrements in oxygen availability under physiological and pathological conditions (15). When pVHL is defective, or oxygen levels are low, HIFα escapes ubiquitylation and accumulates in cells, whereupon it binds to HIFβ and transcriptionally activates genes containing *cis*-acting HIF binding sites referred to as hypoxia response elements (HREs) (15).

## HIF and genotype-phenotype correlations

**Figure 2:**
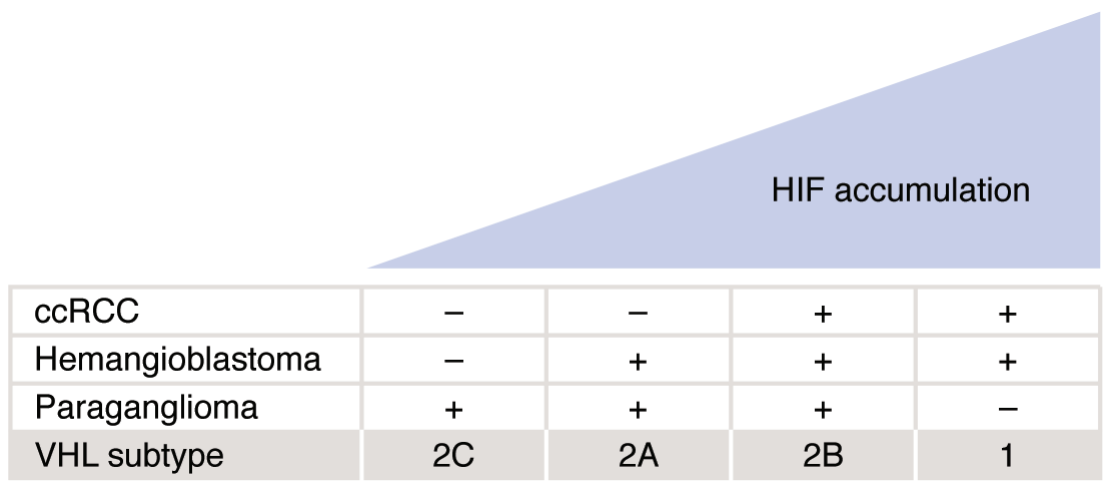
HIF and genotype-phenotype correlations in VHL disease. The degree to which different *VHL* alleles deregulate HIF, at least when tested in preclinical models, correlates with the risk of developing specific types of tumors in VHL disease. *VHL* alleles leading to the highest HIF levels, including true null *VHL* alleles, are associated with a high risk of hemangioblastoma and ccRCC, but not paraganglioma (type 1 VHL disease). *VHL* alleles associated with minimal HIF deregulation are associated with familial paraganglioma with few or no other stigmata of VHL disease (type 2C disease). Progressively higher HIF levels are associated with an increased risk of hemangioblastoma (type 2A disease) and, above a certain threshold, ccRCC (type 2B disease). It is possible that exceedingly high HIF levels actually suppress the development of paraganglioma in type 1 disease.

Mutant *VHL* alleles linked to type 1, type 2A, type 2B, or type 2C VHL disease differ with respect to the degree to which they deregulate HIF, with relative HIF levels being highest in type 1 and lowest in type 2C as follows: type 1 > type 2B > type 2A > type 2C (24) (Figure 2). In fact, type 2C mutants appear fully capable of suppressing HIF, at least when expressed from strong promoters (25, 26). It is possible, however, that such overexpression systems masked subtle defects, including pVHL destabilization, that might have been apparent had these mutants been expressed by the endogenous *VHL* locus. Nonetheless, these observations suggest that complete loss of pVHL, perhaps due to excess HIF activity, is antithetical to paraganglioma development, and either that paraganglioma development reflects a HIF-independent pVHL function or that very subtle HIF dysregulation can cause paraganglioma (see also below).

## HIF and ccRCC

The role of HIF, and particularly HIF2, has been particularly well established in ccRCC. The earliest recognizable pVHL-defective lesions arising in the kidneys of VHL patients demonstrate an increase in HIF and HIF target genes, with the appearance of HIF2 in conjunction with HIF1 in such lesions correlating with worsening cellular atypia and more advanced disease (13, 27). In mouse *VHL^–/–^* ccRCC xenograft experiments, silencing HIF2α using shRNAs or CRISPR/Cas9 suppresses tumor growth whereas forced production of HIF2α, such as through the expression of a non-hydroxylatable HIF2α mutant, bypasses pVHL’s tumor suppressor activity (28–32). In stark contrast, HIF1α constrains *VHL^–/–^* ccRCC growth in such assays (31–34). A caveat is that some *VHL^–/–^* ccRCC lines are not affected by manipulations of HIF2 activity, suggesting they either were never HIF2-dependent or became HIF2-independent in the course of tumor progression in vivo or during cell line creation and passage ex vivo (35, 36). Interestingly, a polymorphism in the HIF2α locus has been linked to risk of ccRCC in humans (37).

## HIF and paraganglioma

As described above, type 2C pVHL mutants, when overexpressed, retain the ability to suppress HIF. This led to the suggestion that pVHL has HIF-independent functions that repress paraganglioma development. In this regard, wild-type pVHL, but not type 2C pVHL mutants, can suppress aPKC activity and thereby suppress JunB (38). The inappropriate accumulation of JunB can repress c-Jun, which plays a critical role in the culling of excess sympathetic neuroblasts during development. This lack of culling might contribute to the development of paragangliomas, which arise from sympathetic/adrenal-lineage cells. On the other hand, loss-of-function EglN1 and gain-of-function HIF2α mutations have also been identified, albeit rarely, in paragangliomas (5). This suggests that pVHL mutant paragangliomas, including those bearing type 2 mutations, are driven by HIF2α and that the failure to demonstrate a defect in HIF regulation by type 2C mutants was technical. It remains possible, however, that derepression of either HIF or JunB (or perhaps some other HIF-independent pVHL substrate) can cause paragangliomas.

## HIF and hemangioblastoma

Hemangioblastomas are seen in both type 2A and type 2B VHL disease, while ccRCC is, by definition, associated exclusively with the latter. Type 2B pVHL mutants lead to higher HIF levels than type 2A mutants, suggesting that hemangioblastoma development requires less HIF activation than does ccRCC development (39). Early work showed that forced expression of VEGF in the mouse causes the development of hemangioblastoma-like lesions, strongly suggesting that deregulated HIF, and hence VEGF, contributes to the pathogenesis of these tumors (40). Inactivation of VHL in various mouse tissues also causes vascular proliferations that very loosely resemble hemangioblastomas (see below).

## Cooperating mutations in ccRCC

VHL inactivation causes renal cysts in mice and humans, but is not sufficient to cause ccRCC. Both hereditary (VHL disease) and sporadic ccRCCs frequently harbor loss of chromosome 3p, gain of chromosome 5q, and loss of chromosome 14q, with *VHL* loss serving as the initiating or “truncal” event (41). Chromosome 3p harbors four bona fide ccRCC tumor suppressor genes: *VHL*, *BAP1*, *PBRM1*, and *SETD2* (41). Most ccRCCs have suffered biallelic loss of *BAP1*, *PBRM1*, or, rarely, both (41). PRBM1 loss amplifies the deregulation of HIF in ccRCC (42, 43). Chromosome 5q harbors several candidate ccRCC proto-oncogenes, including *SQSTM1*, which encodes the p62 protein that has been implicated in the regulation of autophagy, NRF2 signaling, and mTOR signaling (44). Chromosome 14q harbors several genes that can function to constrain ccRCC growth, including *HIF1A* (34). Intragenic missense mutations have also been described in ccRCCs that affect mTOR signaling, redox stress signaling, or the response to DNA damage (41).

## Mouse models of *VHL^–/–^* ccRCC

*Vhl^–/–^* mouse embryos are not viable, and *Vhl^+/–^* mice are grossly normal (45, 46). In particular, *Vhl^+/–^* mice do not phenocopy humans in the mouse strains examined to date. Targeted biallelic inactivation of *Vhl* in mouse kidneys and liver causes renal cysts and hemangiomas, respectively, apparently driven by HIF2 and not HIF1 (46–50). Similarly, HIF2 appears to drive the increased vascularization noted after biallelic *Vhl* inactivation in skin (50).

Multiple attempts have been made to combine *Vhl* loss with other cooperating genetic events, either by breeding or, more recently, by somatic gene editing with CRISPR/Cas9, in hopes of creating a murine model of human *VHL^–/–^* ccRCC (43, 51–56). These models typically yield small tumors with long latencies and/or introduce mutations, such as p53 mutations, that are not common in primary human *VHL^–/–^* ccRCCs. In most of these models HIF2 dependence has not been firmly established. Notably, some of the recurrent genetic changes in human ccRCC involve chromosomal arms, suggesting the presence of multiple pathogenic targets on those arms that contribute to ccRCC pathogenesis. Therefore it might be challenging to recapitulate these changes with single gene disruptions. In this regard, human ccRCC tumor suppressor genes, including *PBRM1*, *BAP1*, and *SETD2*, are located on chromosome 3p, but their murine orthologs are located on separate chromosomes.

A mouse model of Chuvash polycythemia was made using homologous recombination of embryonic stem cells to create *Vhl^R166W/R166W^* mice (equivalent to *VHL^R200W/R200W^* in humans) (57). As expected based on their human counterparts, these mice develop florid polycythemia but are not at high risk of the tumors seen in VHL disease.

## Medical treatment of VHL-associated neoplasms

The tumors linked to *VHL* inactivation, including ccRCCs, are highly angiogenic, which can now be rationalized based on the knowledge that pVHL regulates HIF-responsive proangiogenic genes such as VEGF. Drugs that inhibit VEGF or one of its key receptors, KDR, have become mainstays of ccRCC treatment. Indeed, eight such drugs are now FDA approved for this indication (58). Nonetheless, not all ccRCC patients respond to VEGF inhibitors, and virtually all ccRCC patients who do initially respond to VEGF inhibitors will eventually progress. There is much less information available with respect to the use of VEGF inhibitors to treat hemangioblastomas. In this setting VEGF inhibitors can sometimes improve symptoms, probably owing to decreased peritumoral edema because of a decrease in VEGF-induced vascular leakiness, but they do not typically cause objective tumor regressions (59, 60). Whether they slow hemangioblastoma growth is hard to assess, as these tumors are slow-growing to begin with.

The preclinical studies described above nominated HIF2 as a potential therapeutic target in ccRCC. Although HIF2 was classically viewed as an “undruggable” transcription factor, Rick Bruick and Kevin Gardner identified a potentially druggable pocket in HIF2α, as well as chemicals that, upon binding to this pocket, induced an allosteric change that prevented HIF2α from binding to its heterodimeric partner ARNT (and hence to DNA) (61–63). These seminal findings enabled the creation of first-in-class small-molecule HIF2 inhibitors by Peloton Therapeutics, which was subsequently acquired by Merck. Such inhibitors are active against HIF2-dependent, pVHL-defective, ccRCC in preclinical models (36, 64, 65). The most advanced of these, belzutifan, was recently FDA approved for VHL disease based on a phase II trial of 61 VHL patients with at least one measurable ccRCC (66). Virtually all the evaluable ccRCCs in this trial measurably shrank when treated with belzutifan, including many that fulfilled RECIST partial response criteria (66). This suggests that all pVHL-defective ccRCCs are initially HIF2 dependent. Gratifyingly, responses were also seen in non-indicator lesions such as hemangioblastomas and pancreatic neuroendocrine tumors (66). Belzutifan has also advanced to phase III trials for sporadic ccRCCs based on very promising phase II data (67) and was also recently reported to be active in the treatment of a paraganglioma caused by a gain-of-function HIF2 mutation (68).

## HIF, endogenous retroviruses, and tumor immunogenicity

ccRCC has historically been viewed as an immunogenic tumor because it occasionally spontaneously regresses and because it sometimes responds to immune modulators such as high-dose interleukin-2, interferon, or, more recently, immune checkpoint blockade (69–71). ccRCCs also are characterized by a high level of effector T cells (72). The cause of this presumed immunogenicity is not clear. In particular, ccRCC does not have the high mutational burden observed in other immunogenic tumors such as malignant melanoma and mismatch repair–deficient colorectal cancer (73).

Some, but not all, studies have suggested a link between the expression of endogenous retroviruses (ERVs) in ccRCCs and their likelihood of responding to immune checkpoint blockade (74–77). Intriguingly, Richard Childs and coworkers treated a series of patients with metastatic ccRCC with allogenic stem cell transplants (78). Almost half of the patients treated in this way exhibited objective tumor regressions, including some who had complete responses. In one complete responder the response was linked to donor T cells that recognized an HLA-bound peptide that was derived from the endogenous retrovirus HERV-E (79, 80). The authors showed that the expression of this ERV was restricted to ccRCC and not observed in other cancers or normal tissues. They further demonstrated that HERV-E expression is driven by HIF2, thus at least partly explaining its deregulation in ccRCC. It will be of interest to determine whether HIF drives the promiscuous expression of additional ERVs and, if so, whether this contributes to the immunogenicity of ccRCC.

## 2-Oxoglutarate–dependent dioxygenases as oxygen sensors

The EglN prolyl hydroxylases belong to a larger superfamily of approximately 70 2-oxoglutarate–dependent (2-OG–dependent) dioxygenases that includes the JmjC domain–containing histone demethylases, the TET DNA hydroxylases, the collagen prolyl hydroxylases, and the FIH1 asparaginyl hydroxylase (81, 82). The EglNs have relatively low oxygen affinities, and are therefore poised to act as oxygen sensors. In contrast, the collagen prolyl hydroxylases have relatively high oxygen affinities, rendering them relatively insensitive to changes in oxygen until cells become virtually anoxic (15). We and others showed that some, but not all, of the JmjC domain–containing histone demethylases have low oxygen affinities and can thereby directly translate changes in oxygen availability into changes in histone methylation and thereby gene expression (83, 84). For example, the two principal H3K27 demethylases are KDM6A and KDM6B. We showed that KDM6A has a low oxygen affinity while KDM6B has a high oxygen affinity. Hypoxia can affect cellular differentiation. We linked the ability of hypoxia to block myogenic differentiation to inhibition of KDM6A rather than to the activation of HIF. These findings have implications for the importance of hypoxia, such as occurs during intrauterine development, in stem cell niches, and in specific organs such as the thymus, in the control of cell fate.

## 2-Hydroxyglutarate: an endogenous 2-OG competitor

Some cancers, such as a subset of gliomas, acute myelogenous leukemias, cholangiocarcinomas, and chondrosarcomas, are caused by mutations in isocitrate dehydrogenase 1 (IDH1) or IDH2 (85). These mutations cause IDH1 and IDH2 to convert 2-OG to 2-hydroxyglutarate (2-HG) rather than to convert isocitrate to 2-OG. 2-HG can accumulate to millimolar levels in IDH mutant tumors and can competitively inhibit various 2-OG–dependent enzymes. For example, IDH mutations appear to cause AML at least partly by inhibiting TET2 (86–88). Many of the enzymes that are inhibited by 2-HG are epigenetic regulators, which led to the concern that blocking 2-HG production would not reverse its transforming activity in a clinically relevant time scale. Fortunately, we found that 2-HG was both necessary and sufficient for transformation in a preclinical model of IDH mutant AML and that its effects were reversible over the course of weeks (88). This galvanized interest in developing drugs that block 2-HG production by mutant IDH1 or IDH2, and two such drugs are now FDA approved for IDH mutant AML.

Surprisingly, we found that 2-HG could stimulate EglN activity, which would explain the low HIF levels observed in IDH mutant gliomas (89). One study suggested that the apparent activation of EglN by 2-HG in vitro reflected contamination with 2-OG (90), but the 2-HG *K_m_* values observed, coupled with the sensitivity of our 2-OG assays, make this extremely unlikely (89). Moreover, HIF levels are characteristically low in IDH mutant gliomas (91), and deletion of HIF1 can augment brain tumor growth in mouse models, suggesting that decreased HIF1 activity contributes to gliomagenesis (92). Importantly, drugs that block 2-HG production have thus far had very modest effects against IDH mutant gliomas in preclinical models and in clinical trials (93–95). In this regard, there is evidence that IDH mutations might act in a “hit and run” manner in glioma, setting in motion a feed-forward cycle that promotes transformation (96, 97).

## HIF agonists and anemia

A number of inhibitors of the EglN prolyl hydroxylases have been developed for the treatment of anemia (Table 1) (98–100). These agents offer an oral alternative to parenteral erythropoietin (EPO) and can also induce red blood cell production in certain EPO-refractory conditions, such as anemia of chronic disease linked to high hepcidin levels. The most advanced of these, roxadustat, has been approved in multiple countries, including China, Japan, South Korea, Chile, and the European Union, for the treatment of anemia caused by chronic kidney disease. Daprodustat is currently approved in Japan and is awaiting approval in Europe. Vadadustat is approved in Japan. The US FDA did not approve roxadustat and vadadustat owing to safety concerns, including a possible increased risk of thrombosis in patients. Whether this risk can be mitigated by titration of drug doses to correct anemia more slowly remains to be determined; this would require additional clinical trials (and perhaps real-world data from post-approval countries).

## HIF and tissue protection

Pharmacological or genetic activation of HIF1α, such as through inactivation of EglN1, protects against tissue damage in models of acute cardiac, cerebral, and renal ischemia (101–107). These acute effects likely reflect acute changes in metabolism that help to preserve ATP under hypoxia conditions, both by promoting a switch from oxidative phosphorylation to anaerobic glycolysis and by reducing ATP-consuming processes such as macromolecule synthesis. Chronic HIF activation, however, can lead to deleterious changes. For example, chronic HIF1α activation in the heart, such as through the cardiac-specific inactivation of EglN family members or cardiac-specific expression of a stabilized version of HIF1α, causes a dilated cardiomyopathy (108, 109). These changes correlate with a loss of mitochondria, likely due to mitochondrial autophagy (“mitophagy”) stimulated by the HIFα-responsive gene product BNIP3 (110). These findings might have implications for chronic ischemic cardiomyopathy in humans, which is usually linked to coronary artery disease. It has been appreciated for decades that the degree of cardiac function in ischemic cardiomyopathy is often out of proportion to the degree of heart muscle destruction caused by prior myocardial infarctions. Collectively, these results suggest that HIF activation is beneficial in the setting of acute ischemia, but can become deleterious when persistent over months or years.

## Remote ischemic protection

Tissues that survive a transient ischemic insult are temporarily partially protected from subsequent ischemic insults, a phenomenon referred to as “ischemic preconditioning.” Ischemic preconditioning is likely due, at least partly, to HIF-dependent metabolic changes that persist after the initial ischemic insult has resolved. “Remote ischemic preconditioning” (RIPC) refers to the phenomenon whereby a tissue that survives an ischemic insult can also protect other tissues and organs at a distance. RIPC has been demonstrated in a variety of laboratory models, but has not been highly reproducible or robust when studied in human clinical trials. For example, two large randomized clinical trials failed to show a clinical benefit when blood pressure cuffs were repeatedly overinflated in order to cause limb ischemia prior to elective heart surgery (111, 112). We reasoned that understanding the mechanism of RIPC in the laboratory might allow one to revisit the possibility of harnessing RIPC for clinical benefit in humans. One consequence of ischemia is to inhibit EglN activity. Accordingly, we created mice in which EglN1 was specifically inactivated in the skeletal muscle before experimental myocardial infarctions (103). We discovered that acutely inactivating EglN1 in skeletal muscle decreased heart muscle damage following experimental myocardial infarctions. RIPC has been hypothesized to involve either neural or hormonal mechanisms. In parabiosis experiments we confirmed that the RIPC in our mouse models involved a circulating factor, which we later identified as kynurenic acid (KynA) (103). KynA had been shown to confer ischemic protection in other models, but its mechanism of action was unknown. In this regard, a number of potential KynA receptors have been reported in the literature (113–119). We showed that ischemic protection by KynA reflects its ability to activate the orphan receptor GPR35 (120). Once bound to KynA, GPR35 internalizes to mitochondria where, in an ATPIF1-dependent manner, it promotes the dimerization of ATP synthase and thereby prevents futile ATP consumption by ATP synthase under hypoxic conditions (120).

## pVHL, protacs, and molecular glues

Ray Deshaies and Craig Crews pioneered the concept of targeting specific proteins for degradation using bifunctional chemicals that would simultaneously bind to a ubiquitin ligase and the target protein of interest, essentially acting as molecular matchmakers (121, 122). They referred to such molecules as proteolysis-targeting chimeras, or protacs. Early protacs tended to be large chemicals with suboptimal medicinal chemistry properties, although several have now advanced to clinical trials.

In the late 1990s the infamous teratogen thalidomide was found, through clinical serendipity, to be highly active in the treatment of the B cell malignancy multiple myeloma. This sparked interest in identifying the relevant protein target(s) of thalidomide. In 2010, Hiroshi Handa and coworkers showed that thalidomide could bind to cereblon, which is the substrate recognition subunit of a ubiquitin ligase (123). Moreover, they provided preliminary evidence that thalidomide was a cereblon antagonist (123).

It was subsequently reported that some myeloma patients who relapsed after initially responding to thalidomide (or related drugs such as lenalidomide) had acquired myeloma cells that no longer produced cereblon (124, 125). Conversely, high levels of cereblon appeared to increase the likelihood of responding to thalidomide-like drugs, now referred to as immunomodulatory imide drugs (IMiDs) (126, 127). The former observation suggested to us and others that the killing of myeloma cells by IMiDs was likely to be due, not to cereblon inhibition, but rather to a neomorphic activity of cereblon once bound to an IMiD. Our group, Ben Ebert’s group, and a group at Celgene Corp. went on to show that the cereblon ubiquitin ligase, once bound to an IMiD, acquires the ability to polyubiquitylate, and hence earmark for destruction, the IKZF1 and IKZF3 transcription factors, which play critical roles in myeloma maintenance (128–130). Shortly thereafter it was reported that the SALL4 transcription factor was likely the neomorphic target responsible for the limb defects associated with thalidomide use during pregnancy (131, 132).

The IMiDs are more “drug-like” than earlier protacs and yet act through the same mechanism envisioned for protacs. As IKZF1 and IKZF3 would classically be viewed as undruggable, this discovery galvanized interest in targeting undruggable proteins with small-molecule degraders, as did the discovery of other bifunctional “molecular glues” such as indisulam, which targets the RBM39 splicing factor by recruitment of DCAF15 (133, 134), and CR8 and HQ461, which target cyclin K by recruitment of CUL4 and DDB1 (135, 136). Moreover, it was quickly established that IMiDs could be chemically modified to change their target specificity (137, 138).

As described above, cereblon loss offers a rapid path to resistance to cereblon-based molecular glues such as the IMiDs. Loss of pVHL confers fitness disadvantage in most cell types examined to date, which likely contributes to the narrow range of tumor types linked to *VHL* mutations. Partly for this reason, a number of pVHL-based protacs and molecular glues have now been developed (121).

## Conclusions and future directions

My mentor, the late David M. Livingston (139), taught me and his other mentees that every good experiment starts with the question it is aimed at addressing. Studying the von Hippel–Lindau tumor suppressor gene shed light on a number of questions: What are the earliest steps in the development of ccRCC, and how should we treat these tumors based on that knowledge? How do tumors regulate angiogenesis, which is a conspicuous feature of VHL-associated tumors, and, more broadly, how do cells and tissues sense oxygen and couple that information to changes in gene expression? The *VHL* gene product, pVHL, proved to be a key node in the oxygen sensing mechanism, targeting the HIF transcription factor for destruction when oxygen is plentiful. Drugs that inhibit HIF2, and HIF-responsive gene products such as VEGF, are now cornerstones of kidney cancer therapy, while HIF agonists, which block oxygen-dependent prolyl hydroxylation of the HIFα subunits, appear promising for the treatment of anemia and ischemia.

A number of questions and mysteries remain. For example, it remains unclear why *VHL* loss is intimately linked to ccRCC, but not other common epithelial cancers. It is potentially relevant in this regard that mammalian kidneys are hypoxic at rest, which might lead to epigenetic changes that allow certain renal cells to proliferate (for example, in response to injury) in a hypoxic environment. Consistent with this idea, HIF lowers cyclin D1 levels and proliferation in many cell types, but increases cyclin D1 and proliferation in the cells capable of giving rise to ccRCC (140). Nor do we completely understand the genotype-phenotype correlations in VHL disease, although the degree of HIF dysregulation almost certainly plays a role here.

It is also unclear how HIF1 and HIF2, which appear to oppose one another with respect to ccRCC proliferation, achieve their paralog-specific effects. Prior reports suggest that this is achieved, at least in part, through paralog-specific binding to specific HIF response elements (141–143). These initial reports, however, might have been confounded by technical factors such as the use of different antibodies or reliance on overexpression systems.

We do not fully understand how, mechanistically, stereotypical non-allelic mutations, such as of *BAP1* and *SETD2*, cooperate with *VHL* loss to cause ccRCC. Nor do we know whether these mutations play roles in tumor maintenance, as opposed to tumor initiation and progression, and whether they engender any specific therapeutic vulnerabilities.

It is not known why the response to VEGF inhibitors and, based on early data, HIF2 inhibitors is variable in ccRCC. With respect to the latter, the percentage of ccRCCs exhibiting measurable tumor shrinkage is much higher in the setting of VHL disease patients whose ccRCCs were previously untreated than in the setting of metastatic disease patients who had been heavily pretreated. This suggests that all ccRCCs are initially HIF2 dependent, but can evolve toward HIF2 independence over time under the selection pressure created by standard-of-care agents such as VEGF inhibitors and immune checkpoint inhibitors.

Our knowledge of the effects of HIF on the immune system is incomplete. This knowledge might influence the outcome of combining HIF2 inhibitors with other anticancer drugs and could prove valuable in exploring the therapeutic utility of HIF agonists. In this regard, we are just beginning to understand the benefits and risks of acutely or chronically inactivating HIF for the treatment of anemia and other disorders such as ischemic diseases. It is perhaps noteworthy that the most advanced HIF agonists inhibit the EglN prolyl hydroxylases, but not the FIH1 asparaginyl hydroxylase (100). Accordingly, these drugs can induce EPO without inducing VEGF, which relies on the FIH1-responsive HIF1α C-terminal transactivation domain in most tissues (144). This was initially viewed as fortuitous, insofar as it was feared VEGF would induce angiogenesis and possibly stimulate latent tumors. On the other hand, there is no evidence that systemic (rather than focal) VEGF induces angiogenesis, presumably because an effective gradient to stimulate endothelial cells is not established, and a recent study showed that increasing systemic VEGF levels in adult mice caused tissue rejuvenation and increased lifespan (145). It is also theoretically possible that a slight increase in VEGF would improve endothelial cell health and reduce, for example, the risk of thrombosis, especially as decreasing VEGF clearly has the opposite effect (146). Combined inhibition of EglN and FIH1 should more faithfully mimic the effects of true hypoxia, such as life at very high altitude. It would therefore be interesting to test combined EglN1 and FIH1 inhibition preclinically and clinically.

## Figures and Tables

**Figure 1:**
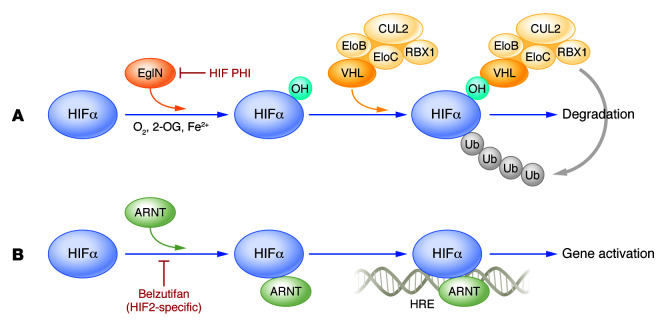
Pharmacological control of HIF. (**A**) In the presence of the cofactors oxygen and reduced iron, the EglN prolyl hydroxylases hydroxylate one (or both) of two prolyl residues in HIFα (for simplicity, only one hydroxylation event is depicted). Prolyl-hydroxylated HIFα is recognized by a ubiquitin ligase that uses pVHL as the substrate recognition module, leading HIFα to be polyubiquitylated and destroyed by the proteasome. HIF prolyl hydroxylase inhibitors (HIF PHIs) prevent the hydroxylation of HIF by inhibiting the EglNs by competing with 2-OG or iron. (**B**) HIFα binds to its partner protein ARNT. This complex can then recognize specific genomic DNA binding sites (hypoxia response elements [HREs]) and activate transcription. Belzutifan binds specifically to HIF2α and induces an allosteric change such that it can no longer bind to ARNT and hence to DNA. EloB, elongin B; EloC, elongin C; CUL2, cullin 2; RBX1, RING box protein 1.

**Table 1:**
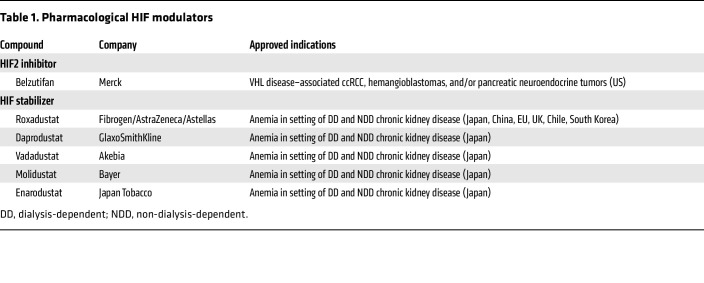
Pharmacological HIF modulators

## Version 1 09/15/2022

Electronic publication

## References

[1] Maher E, Kaelin WG (1997). von Hippel-Lindau disease. Medicine.

[2] Siegel RL (2021). Cancer statistics, 2021. CA Cancer J Clin.

[3] Kaelin WG (2002). Molecular basis of the VHL hereditary cancer syndrome. Nat Rev Cancer.

[4] Kim WY, Kaelin WG (2004). Role of VHL gene mutation in human cancer. J Clin Oncol.

[5] Dahia PL (2017). Pheochromocytomas and paragangliomas, genetically diverse and minimalist, all at once!. Cancer Cell.

[6] Shankar GM (2014). Sporadic hemangioblastomas are characterized by cryptic VHL inactivation. Acta Neuropathol Commun.

[7] Iliopoulos O (1998). pVHL19 is a biologically active product of the von Hippel-Lindau gene arising from internal translation initiation. Proc Natl Acad Sci U S A.

[8] Blankenship C (1999). Alternate choice of initiation codon produces a biologically active product of the von Hippel Lindau gene with tumor suppressor activity. Oncogene.

[9] Schoenfeld A (1998). A second major native von Hippel-Lindau gene product, initiated from an internal translation start site, functions as a tumor suppressor. Proc Natl Acad Sci U S A.

[10] Iliopoulos O (1995). Tumor suppression by the human von Hippel-Lindau gene product. Nat Med.

[11] Lee S (1999). Transcription-dependent nuclear-cytoplasmic trafficking is required for the function of the von Hippel-Lindau tumor suppressor protein. Mol Cell Biol.

[12] Lee S (1996). Nuclear/cytoplasmic localization of the von Hippel-Lindau tumor suppressor gene product is determined by cell density. Proc Natl Acad Sci U S A.

[13] Guo J (2016). pVHL suppresses kinase activity of Akt in a proline-hydroxylation-dependent manner. Science.

[14] Yang H (2007). pVHL acts as an adaptor to promote the inhibitory phosphorylation of the NF-kappaB agonist Card9 by CK2. Mol Cell.

[15] Kaelin WG, Ratcliffe PJ (2008). Oxygen sensing by metazoans: the central role of the HIF hydroxylase pathway. Mol Cell.

[16] Ivan M (2001). HIFalpha targeted for VHL-mediated destruction by proline hydroxylation: implications for O_2_ sensing. Science.

[17] Ivan M (2002). Biochemical purification and pharmacological inhibition of a mammalian prolyl hydroxylase acting on hypoxia-inducible factor. Proc Natl Acad Sci U S A.

[18] Jaakkola P (2001). Targeting of HIF-alpha to the von Hippel-Lindau ubiquitylation complex by O_2_-regulated prolyl hydroxylation. Science.

[19] Epstein A (2001). C. elegans EGL-9 and mammalian homologs define a family of dioxygenases that regulate HIF by prolyl hydroxylation. Cell.

[20] Yu F (2001). HIF-1alpha binding to VHL is regulated by stimulus-sensitive proline hydroxylation. Proc Natl Acad Sci U S A.

[21] Bruick R, McKnight S (2001). A conserved family of prolyl-4-hydroxylases that modify HIF. Science.

[22] Berra E (2003). HIF prolyl-hydroxylase 2 is the key oxygen sensor setting low steady-state levels of HIF-1alpha in normoxia. EMBO J.

[23] Minamishima YA (2009). A feedback loop involving the Phd3 prolyl hydroxylase tunes the mammalian hypoxic response in vivo. Mol Cell Biol.

[24] Kaelin WG (2008). The von Hippel-Lindau tumour suppressor protein: O2 sensing and cancer. Nat Rev Cancer.

[25] Hoffman MA (2001). von Hippel-Lindau protein mutants linked to type 2C VHL disease preserve the ability to downregulate HIF. Hum Mol Genet.

[26] Clifford S (2001). Contrasting effects on HIF-1alpha regulation by disease-causing pVHL mutations correlate with patterns of tumourigenesis in von Hippel-Lindau disease. Hum Mol Genet.

[27] Mandriota SJ (2002). HIF activation identifies early lesions in VHL kidneys: evidence for site-specific tumor suppressor function in the nephron. Cancer Cell.

[28] Kondo K (2003). Inhibition of HIF2alpha is sufficient to suppress pVHL-defective tumor growth. PLoS Biol.

[29] Kondo K (2002). Inhibition of HIF is necessary for tumor suppression by the von Hippel-Lindau protein. Cancer Cell.

[30] Zimmer M (2004). Inhibition of hypoxia-inducible factor is sufficient for growth suppression of VHL^-/-^ tumors. Mol Cancer Res.

[31] Raval RR (2005). Contrasting properties of hypoxia-inducible factor 1 (HIF-1) and HIF-2 in von Hippel-Lindau-associated renal cell carcinoma. Mol Cell Biol.

[32] Gordan JD (2007). HIF-2alpha promotes hypoxic cell proliferation by enhancing c-myc transcriptional activity. Cancer Cell.

[33] Maranchie JK (2002). The contribution of VHL substrate binding and HIF1-alpha to the phenotype of VHL loss in renal cell carcinoma. Cancer Cell.

[34] Shen C (2011). Genetic and functional studies implicate HIF1α as a 14q kidney cancer suppressor gene. Cancer Discov.

[35] Stransky LA (2022). Sensitivity of *VHL* mutant kidney cancers to HIF2 inhibitors does not require an intact p53 pathway. Proc Natl Acad Sci U S A.

[36] Cho H (2016). On-target efficacy of a HIF2alpha antagonist in preclinical kidney cancer models. Nature.

[37] Purdue MP (2011). Genome-wide association study of renal cell carcinoma identifies two susceptibility loci on 2p21 and 11q13.3. Nat Genet.

[38] Lee S (2005). Neuronal apoptosis linked to EglN3 prolyl hydroxylase and familial pheochromocytoma genes: developmental culling and cancer. Cancer Cell.

[39] Li L (2007). Hypoxia-inducible factor linked to differential kidney cancer risk seen with type 2A and type 2B VHL mutations. Mol Cell Biol.

[40] Benjamin LE, Keshet E (1997). Conditional switching of vascular endothelial growth factor (VEGF) expression in tumors: induction of endothelial cell shedding and regression of hemangioblastoma-like vessels by VEGF withdrawal. Proc Natl Acad Sci U S A.

[42] Gao W (2017). Inactivation of the PBRM1 tumor suppressor gene amplifies the HIF-response in VHL^-/-^ clear cell renal carcinoma. Proc Natl Acad Sci U S A.

[43] Nargund AM (2017). The SWI/SNF protein PBRM1 restrains VHL-loss-driven clear cell renal cell carcinoma. Cell Rep.

[44] Li L (2013). SQSTM1 is a pathogenic target of 5q copy number gains in kidney cancer. Cancer Cell.

[45] Gnarra J (1997). Defective placental vasculogenesis causes embryonic lethality in VHL-deficient mice. Proc Natl Acad Sci U S A.

[46] Ma W (2003). Hepatic vascular tumors, angiectasis in multiple organs, and impaired spermatogenesis in mice with conditional inactivation of the VHL gene. Cancer Res.

[47] Rankin EB (2006). Renal cyst development in mice with conditional inactivation of the von Hippel-Lindau tumor suppressor. Cancer Res.

[48] Rankin EB (2005). Inactivation of the arylhydrocarbon receptor nuclear translocator (Arnt) suppresses von Hippel-Lindau disease-associated vascular tumors in mice. Mol Cell Biol.

[49] Rankin EB (2008). Hypoxia-inducible factor-2 regulates vascular tumorigenesis in mice. Oncogene.

[50] Kim WY (2006). Failure to prolyl hydroxylate hypoxia-inducible factor alpha phenocopies VHL inactivation in vivo. EMBO J.

[51] Wang SS (2014). Bap1 is essential for kidney function and cooperates with Vhl in renal tumorigenesis. Proc Natl Acad Sci U S A.

[52] Schonenberger D (2016). Formation of renal cysts and tumors in Vhl/Trp53-deficient mice requires HIF1α and HIF2α. Cancer Res.

[53] Fu L (2011). Generation of a mouse model of Von Hippel-Lindau kidney disease leading to renal cancers by expression of a constitutively active mutant of HIF1α. Cancer Res.

[54] Hou W, Ji Z (2018). Generation of autochthonous mouse models of clear cell renal cell carcinoma: mouse models of renal cell carcinoma. Exp Mol Med.

[55] Bailey ST (2017). MYC activation cooperates with Vhl and Ink4a/Arf loss to induce clear cell renal cell carcinoma. Nat Commun.

[56] Gu YF (2017). Modeling renal cell carcinoma in mice: *Bap1* and *Pbrm1* inactivation drive tumor grade. Cancer Discov.

[57] Hickey MM (2007). von Hippel-Lindau mutation in mice recapitulates Chuvash polycythemia via hypoxia-inducible factor-2alpha signaling and splenic erythropoiesis. J Clin Invest.

[58] Choueiri TK, Kaelin WG (2020). Targeting the HIF2-VEGF axis in renal cell carcinoma. Nat Med.

[59] Aiello LP (2002). Rapid and durable recovery of visual function in a patient with von Hippel-Lindau syndrome after systemic therapy with vascular endothelial growth factor receptor inhibitor su5416. Ophthalmology.

[60] Jonasch E (2011). Pilot trial of sunitinib therapy in patients with von Hippel-Lindau disease. Ann Oncol.

[61] Rogers JL (2013). Development of inhibitors of the PAS-B domain of the HIF-2α transcription factor. J Med Chem.

[62] Scheuermann TH (2013). Allosteric inhibition of hypoxia inducible factor-2 with small molecules. Nat Chem Biol.

[63] Scheuermann TH (2009). Artificial ligand binding within the HIF2alpha PAS-B domain of the HIF2 transcription factor. Proc Natl Acad Sci U S A.

[64] Chen W (2016). Targeting renal cell carcinoma with a HIF-2 antagonist. Nature.

[65] Wallace EM (2016). A small-molecule antagonist of HIF2α is efficacious in preclinical models of renal cell carcinoma. Cancer Res.

[66] Jonasch E (2021). Belzutifan for renal cell carcinoma in von Hippel-Lindau Disease. N Engl J Med.

[67] Choueiri TK (2021). Inhibition of hypoxia-inducible factor-2alpha in renal cell carcinoma with belzutifan: a phase 1 trial and biomarker analysis. Nat Med.

[68] Kamihara J (2021). Belzutifan, a potent HIF2α inhibitor, in the Pacak-Zhuang syndrome. N Engl J Med.

[69] Xu W (2020). Checkpoint inhibitor immunotherapy in kidney cancer. Nat Rev Urol.

[70] McDermott DF, Atkins MB (2013). Immune therapy for kidney cancer: a second dawn?. Semin Oncol.

[71] McDermott DF, Atkins MB (2004). Application of IL-2 and other cytokines in renal cancer. Expert Opin Biol Ther.

[72] Rooney MS (2015). Molecular and genetic properties of tumors associated with local immune cytolytic activity. Cell.

[73] Lawrence MS (2013). Mutational heterogeneity in cancer and the search for new cancer-associated genes. Nature.

[74] Panda A (2018). Endogenous retrovirus expression is associated with response to immune checkpoint blockade in clear cell renal cell carcinoma. JCI Insight.

[75] Smith CC (2018). Endogenous retroviral signatures predict immunotherapy response in clear cell renal cell carcinoma. J Clin Invest.

[76] Au L (2021). Determinants of anti-PD-1 response and resistance in clear cell renal cell carcinoma. Cancer Cell.

[77] de Cubas AA (2020). DNA hypomethylation promotes transposable element expression and activation of immune signaling in renal cell cancer. JCI Insight.

[78] Takahashi Y (2008). Regression of human kidney cancer following allogeneic stem cell transplantation is associated with recognition of an HERV-E antigen by T cells. J Clin Invest.

[79] Cherkasova E (2016). Detection of an immunogenic HERV-E envelope with selective expression in clear cell kidney cancer. Cancer Res.

[80] Cherkasova E (2011). Inactivation of the von Hippel-Lindau tumor suppressor leads to selective expression of a human endogenous retrovirus in kidney cancer. Oncogene.

[81] Aravind L, Koonin EV (2001). The DNA-repair protein AlkB, EGL-9, and leprecan define new families of 2-oxoglutarate- and iron-dependent dioxygenases. Genome Biol.

[82] Losman JA (2020). 2-Oxoglutarate-dependent dioxygenases in cancer. Nat Rev Cancer.

[83] Chakraborty AA (2019). Histone demethylase KDM6A directly senses oxygen to control chromatin and cell fate. Science.

[84] Batie M (2019). Hypoxia induces rapid changes to histone methylation and reprograms chromatin. Science.

[85] Losman JA, Kaelin WG (2013). What a difference a hydroxyl makes: mutant IDH, (R)-2-hydroxyglutarate, and cancer. Genes Dev.

[86] Ko M (2011). Ten-Eleven-Translocation 2 (TET2) negatively regulates homeostasis and differentiation of hematopoietic stem cells in mice. Proc Natl Acad Sci U S A.

[87] Figueroa ME (2010). Leukemic IDH1 and IDH2 mutations result in a hypermethylation phenotype, disrupt TET2 function, and impair hematopoietic differentiation. Cancer Cell.

[88] Losman JA (2013). (R)-2-hydroxyglutarate is sufficient to promote leukemogenesis and its effects are reversible. Science.

[89] Koivunen P (2012). Transformation by the (R)-enantiomer of 2-hydroxyglutarate linked to EGLN activation. Nature.

[90] Tarhonskaya H (2014). Non-enzymatic chemistry enables 2-hydroxyglutarate-mediated activation of 2-oxoglutarate oxygenases. Nat Commun.

[91] Williams SC (2011). R132H-mutation of isocitrate dehydrogenase-1 is not sufficient for HIF-1α upregulation in adult glioma. Acta Neuropathol.

[92] Blouw B (2003). The hypoxic response of tumors is dependent on their microenvironment. Cancer Cell.

[93] Tateishi K (2015). Extreme vulnerability of IDH1 mutant cancers to NAD+ depletion. Cancer Cell.

[94] Mellinghoff IK (2021). Vorasidenib, a dual inhibitor of mutant IDH1/2, in recurrent or progressive glioma: results of a first-in-human phase I trial. Clin Cancer Res.

[95] Rohle D (2013). An inhibitor of mutant IDH1 delays growth and promotes differentiation of glioma cells. Science.

[96] Johannessen TA (2016). Rapid conversion of mutant IDH1 from driver to passenger in a model of human gliomagenesis. Mol Cancer Res.

[97] Flavahan WA (2016). Insulator dysfunction and oncogene activation in IDH mutant gliomas. Nature.

[98] Gupta N, Wish JB (2017). Hypoxia-inducible factor prolyl hydroxylase inhibitors: a potential new treatment for anemia in patients with CKD. Am J Kidney Dis.

[99] Joharapurkar AA (2018). Prolyl hydroxylase inhibitors: a breakthrough in the therapy of anemia associated with chronic diseases. J Med Chem.

[100] Yeh TL (2017). Molecular and cellular mechanisms of HIF prolyl hydroxylase inhibitors in clinical trials. Chem Sci.

[101] Eckle T (2008). Hypoxia-inducible factor-1 is central to cardioprotection: a new paradigm for ischemic preconditioning. Circulation.

[102] Cai Z (2008). Complete loss of ischaemic preconditioning-induced cardioprotection in mice with partial deficiency of HIF-1 alpha. Cardiovasc Res.

[103] Olenchock BA (2016). EGLN1 inhibition and rerouting of alpha-ketoglutarate suffice for remote ischemic protection. Cell.

[104] Kant R (2008). Remote renal preconditioning-induced cardioprotection: a key role of hypoxia inducible factor-prolyl 4-hydroxylases. Mol Cell Biochem.

[105] Ratan RR (2007). Harnessing hypoxic adaptation to prevent, treat, and repair stroke. J Mol Med (Berl).

[106] Harten SK (2010). Prolyl hydroxylase domain inhibitors: a route to HIF activation and neuroprotection. Antioxid Redox Signal.

[107] Maxwell PH, Eckardt KU (2016). HIF prolyl hydroxylase inhibitors for the treatment of renal anaemia and beyond. Nat Rev Nephrol.

[108] Moslehi J (2010). Loss of hypoxia-inducible factor prolyl hydroxylase activity in cardiomyocytes phenocopies ischemic cardiomyopathy. Circulation.

[109] Bekeredjian R (2010). Conditional HIF-1alpha expression produces a reversible cardiomyopathy. PLoS One.

[110] Bellot G (2009). Hypoxia-induced autophagy is mediated through hypoxia-inducible factor induction of BNIP3 and BNIP3L via their BH3 domains. Mol Cell Biol.

[111] Meybohm P (2015). A multicenter trial of remote ischemic preconditioning for heart surgery. N Engl J Med.

[112] Hausenloy DJ (2015). Remote ischemic preconditioning and outcomes of cardiac surgery. N Engl J Med.

[113] Hilmas C (2001). The brain metabolite kynurenic acid inhibits alpha7 nicotinic receptor activity and increases non-alpha7 nicotinic receptor expression: physiopathological implications. J Neurosci.

[114] Stone TW (2007). Kynurenic acid blocks nicotinic synaptic transmission to hippocampal interneurons in young rats. Eur J Neurosci.

[115] Mok MH (2009). Electrophysiological characterisation of the actions of kynurenic acid at ligand-gated ion channels. Neuropharmacology.

[116] Resta F (2016). Kynurenic acid and zaprinast induce analgesia by modulating HCN channels through GPR35 activation. Neuropharmacology.

[117] Wang J (2006). Kynurenic acid as a ligand for orphan G protein-coupled receptor GPR35. J Biol Chem.

[118] DiNatale BC (2010). Kynurenic acid is a potent endogenous aryl hydrocarbon receptor ligand that synergistically induces interleukin-6 in the presence of inflammatory signaling. Toxicol Sci.

[119] Wang D (2021). Functional metabolomics reveal the role of AHR/GPR35 mediated kynurenic acid gradient sensing in chemotherapy-induced intestinal damage. Acta Pharm Sin B.

[120] Wyant GA (2022). Mitochondrial remodeling and ischemic protection by G-protein coupled receptor 35 agonists. Science.

[121] Schapira M (2019). Targeted protein degradation: expanding the toolbox. Nat Rev Drug Discov.

[122] Burslem GM, Crews CM (2020). Proteolysis-targeting chimeras as therapeutics and tools for biological discovery. Cell.

[123] Ito T (2010). Identification of a primary target of thalidomide teratogenicity. Science.

[124] Lopez-Girona A (2012). Cereblon is a direct protein target for immunomodulatory and antiproliferative activities of lenalidomide and pomalidomide. Leukemia.

[125] Zhu YX (2011). Cereblon expression is required for the antimyeloma activity of lenalidomide and pomalidomide. Blood.

[126] Heintel D (2013). High expression of cereblon (CRBN) is associated with improved clinical response in patients with multiple myeloma treated with lenalidomide and dexamethasone. Br J Haematol.

[127] Broyl A (2013). High cereblon expression is associated with better survival in patients with newly diagnosed multiple myeloma treated with thalidomide maintenance. Blood.

[128] Lu G (2014). The myeloma drug lenalidomide promotes the cereblon-dependent destruction of Ikaros proteins. Science.

[129] Kronke J (2014). Lenalidomide causes selective degradation of IKZF1 and IKZF3 in multiple myeloma cells. Science.

[130] Gandhi AK (2014). Immunomodulatory agents lenalidomide and pomalidomide co-stimulate T cells by inducing degradation of T cell repressors Ikaros and Aiolos via modulation of the E3 ubiquitin ligase complex CRL4^CRBN^. Br J Haematol.

[131] Matyskiela ME (2018). SALL4 mediates teratogenicity as a thalidomide-dependent cereblon substrate. Nat Chem Biol.

[132] Donovan KA (2018). Thalidomide promotes degradation of SALL4, a transcription factor implicated in Duane Radial Ray syndrome. Elife.

[133] Han T (2017). Anticancer sulfonamides target splicing by inducing RBM39 degradation via recruitment to DCAF15. Science.

[134] Uehara T (2017). Selective degradation of splicing factor CAPERα by anticancer sulfonamides. Nat Chem Biol.

[135] Lv L (2020). Discovery of a molecular glue promoting CDK12-DDB1 interaction to trigger cyclin K degradation. Elife.

[136] Slabicki M (2020). The CDK inhibitor CR8 acts as a molecular glue degrader that depletes cyclin K. Nature.

[137] Winter GE (2015). Phthalimide conjugation as a strategy for in vivo target protein degradation. Science.

[138] Lu J (2015). Hijacking the E3 ubiquitin ligase cereblon to efficiently target BRD4. Chem Biol.

[139] Kaelin WG (2021). David M. Livingston (1941–2021). Cell.

[140] Bindra RS (2002). VHL-mediated hypoxia regulation of cyclin D1 in renal carcinoma cells. Cancer Res.

[141] Smythies JA (2019). Inherent DNA-binding specificities of the HIF-1α and HIF-2α transcription factors in chromatin. EMBO Rep.

[142] Schodel J (2011). High-resolution genome-wide mapping of HIF-binding sites by ChIP-seq. Blood.

[143] Schodel J (2013). Pan-genomic binding of hypoxia-inducible transcription factors. Biol Chem.

[144] Dayan F (2006). The oxygen sensor factor-inhibiting hypoxia-inducible factor-1 controls expression of distinct genes through the bifunctional transcriptional character of hypoxia-inducible factor-1alpha. Cancer Res.

[145] Grunewald M (2021). Counteracting age-related VEGF signaling insufficiency promotes healthy aging and extends life span. Science.

[146] Touyz RM (2018). Vascular toxicities with VEGF inhibitor therapies-focus on hypertension and arterial thrombotic events. J Am Soc Hypertens.

